# Anti-Inflammatory and Anticoagulative Effects of Paeonol on LPS-Induced Acute Lung Injury in Rats

**DOI:** 10.1155/2012/837513

**Published:** 2012-02-21

**Authors:** Pin-Kuei Fu, Chieh-Liang Wu, Tung-Hu Tsai, Ching-Liang Hsieh

**Affiliations:** ^1^Division of Critical Care and Respiratory Therapy, Department of Internal Medicine, Taichung Veterans General Hospital, Taichung 407, Taiwan; ^2^College of Chinese Medicine, Graduate Institute of Chinese Medical Science, China Medical University, Taichung 40402, Taiwan; ^3^Division of Chest Medicine, Department of Internal Medicine, Taichung Veterans General Hospital, Chiayi Branch, Chiayi 600, Taiwan; ^4^College of Medicine, School of Medicine, China Medical University, Taichung 40402, Taiwan; ^5^Institute of Traditional Medicine, School of Medicine, National Yang-Ming University, Taipei, Taiwan; ^6^Department of Chinese Medicine, China Medical University Hospital, Taichung 40402, Taiwan; ^7^College of Chinese Medicine, Graduate Institute of Acupuncture Science, China Medical University, Taichung 40402, Taiwan; ^8^Acupuncture Research Center, China Medical University, Taichung 40402, Taiwan

## Abstract

Paeonol is an active component of Moutan Cortex Radicis and is widely used as an analgesic, antipyretic, and anti-inflammatory agent in traditional Chinese medicine. We wanted to determine the role of paeonol in treating adult respiratory distress syndrome (ARDS). We established an acute lung injury (ALI) model in Sprague-Dawley rats, which was similar to ARDS in humans, using intratracheal administration of lipopolysaccharide (LPS). The intraperitoneal administration of paeonol successfully reduced histopathological scores and attenuated myeloperoxidase-reactive cells as an index of polymorphonuclear neutrophils infiltration and also reduces inducible nitric oxide synthase expression in the lung tissue, at 16 h after LPS administration. In addition, paeonol reduced proinflammatory cytokines in bronchoalveolar lavage fluid, including tumor-necrosis factor-*α*, interleukin-1*β*, interleukin-6, and plasminogen-activated inhibition factor-1. These results indicated that paeonol successfully attenuates inflammatory and coagulation reactions to protect against ALI.

## 1. Introduction

Acute lung injury (ALI) and acute respiratory distress syndrome (ARDS) are initiated either by direct injury to the lung or by a systemic inflammatory process. Both conditions are characterized by serial pulmonary inflammatory responses of the alveolar-capillary membrane. These responses include polymorphonuclear neutrophils (PMN) accumulation [1, 2], disruption of epithelial integrity, interstitial edema, and leakage of large amounts of protein into the alveolar space [3, 4]. Previous research has used animal models to study the pathophysiologic mechanism of ARDS. The *in vivo* intratracheal (IT) administration of lipopolysaccharide (LPS) has been widely accepted as a clinically relevant animal model of ALI/ARDS [5–7]. The molecular events observed after LPS-induced ALI are local recruitment and activation of PMN [1]; release of proinflammatory cytokines, such as tumor necrosis factor (TNF)-*α*, Interleukine (IL)-1*β*, and IL-6 [8]; the formation of reactive oxygen and nitrogen species [9, 10]. Upregulation of inducible nitric oxide synthase (iNOS) that increased nitric oxide (NO) production plays an important role in mediating lung inflammation, a fact that has been well established in both animal models and humans [11–14]. In addition, activated PMN contributes to an increase in protease activity (such as myeloperoxidase (MPO) and lysozyme activity) and promotes the formation of various oxygen metabolites, finally leading to diffused alveolar matrix damage [2, 15].

High levels of proinflammatory cytokines such as TNF-*α*, IL-1*β*, and IL-6 are released, leading to an inflammatory cascade and triggering pulmonary coagulopathy at the same time [5, 16, 17]. These proinflammatory cytokines may activate the coagulation cascade by stimulating tissue factor (TF) expression and attenuating fibrinolysis by stimulating the release of plasminogen activators inhibitors (PAI). The final stage is fibrin deposition in the airspaces and lung microvasculature [16, 18–21]. Studies regarding pulmonary manifestations in patients suffering from ARDS have found enhanced expression of TF, factor VIIa (FVIIa), and TF-dependent factor X (FX) in bronchoalveolar lavage fluid (BALF) [22–25]. In a large multicenter study, higher levels of PAI-1 (the hallmark of impaired fibrinolysis) showed a synergistic association with ALI/ARDS, resulting in higher mortality rates among such patients [26]. Recently, researchers have become increasingly interested in the interplay between coagulation and inflammation in ALI/ARDS. Pulmonary coagulopathy is now accepted as a new target in the treatment of ALI/ARDS [16, 27].

Moutan cortex Radicis (MC), the root cortex of *Paeonia suffruticosa* Andrews, is widely used as an analgesic, antipyretic, and anti-inflammatory agent in traditional Chinese medicine (TCM) [28, 29]. According to ancient Chinese medicine, this herb is used to regulate human sickness such as eliminating heat, promoting blood flow, and removing blood stasis. Paeonol (20-hydroxy-40-methoxyacetophenone), a major phenolic component of MC, improves blood circulation through its inhibitory effects on both platelet aggregation and blood coagulation [30, 31]. Research also indicated that paeonol could inhibit the expression of cell-surface adhesion molecules [32], proinflammatory cytokines such as TNF-*α*  and IL-1*β*  [33, 34], and iNOS-mediated NO [35] and reactive oxygen species production [34, 35]. In addition, paeonol inhibits the generation of proinflammatory cytokine, and increases the production of IL-10 in carrageenan-evoked thermal hyperalgesia rats [36]. However, the effect of paeonol treatment on proinflammatory cytokines (TNF-*α*, IL-1*β*, and IL-6), iNOS-mediated NO, and illness severity had not been studied in the LPS-induced ALI rat model prior to our research. Furthermore, we wanted to investigate the effect of paeonol regarding anticoagulation and antifibrinolysis after LPS challenge. We studied the effects of paeonol using intraperitoneal (IP) injection in an experimental rat model of ALI (induced by IT instillation of LPS) *in vivo* and attempted to clarify the mechanism involved. 

## 2. Materials and Methods

### 2.1. Preparation of Paeonol from MC

The source of paeonol isolated from the root bark of *Paeonia suffruticosa *and extracted with 95% ethanol (1 : 10, w/v) at 50 °C as described Hsieh et al. (2006) [34]. 

### 2.2. Establishment of LPS-Induced ALI Animal Model

This study used pathogen-free, adult male Sprague-Dawley (SD) rats, weighing around 250 to 300 g each. The rats were housed in standard iron cages with a twelve-hour light/dark cycle in the animal facility of Taichung Veterans General Hospital (TCVGH). The animal experiments were approved by the Animal Study Protocol Review Board of TCVGH and were conducted according to the principles stated in the “*Guide for the Care and Use of Laboratory Animals*.” 

The body weight and rectal temperature (RT) of the rats were checked before the LPS challenge was introduced (0 h). The rats were then lightly anesthetized with inhaled 2% isoflurane (Halocarbon Laboratories Div Halocarbon Products Crop, River Edge, NJ, USA) in 0.5 L/min O_2_, 16 mg/kg LPS (Escherichia coli 055: B5; Sigma Chemical Co., St Louis, MO, USA), and 0.5 mL of phosphate buffer saline (PBS). This mixture was delivered into the lungs of the experimental rats by a Micro-Sprayer and Laryngoscope (Penn-Century, Philadelphia, PA, USA) as previously described [37, 38]. An equal volume of PBS alone was used for the control group. After IT instillation, the rat was placed in a vertical position and rotated for 30 s to let the instillation distribute evenly throughout the lungs. Sixteen hours after inoculation, the rats' RT was measured again, and they were then killed with CO_2_ asphyxiation, as described in our pilot study.

### 2.3. Grouping

A total of 30 SD rats were randomized on a daily basis to a PBS control group or a treatment group. Treatment groups received paeonol resolved in DMSO. The effective dosage of paeonol resolved in DMSO was tested in the range of 10 mg/kg to 50 mg/kg, according to guidelines presented in previous literature [32, 34, 39–41]. The double dose of the effective dosage was also tested to investigate the dosage effectiveness. All agents were administered in IP bolus injections under light sedation with 2% isoflurane within 10 min after instillation of LPS. Control group rats (LPS-DMSO) received IP administered with the same volume of DMSO. In contrast, the control group (PBS-DMSO) was IT challenged with a PBS solution lacking LPS. At 16 h after inoculation, all rats were sacrificed with CO_2_ asphyxiation, and their lungs were removed.

In summary, the rats were randomly divided into five groups comprising 6 rats each, as follows

1. PBS-DMSO group: IT-challenged PBS and IP DMSO,
2. PBS-paeonol group: IT PBS and IP paeonol 25 mg/kg,
3. LPS-DMSO group: IT LPS and IP DMSO,
4. LPS-paeonol-25 group: IT LPS and IP paeonol 25 mg/kg, and
5. LPS-paeonol-50 group: IT LPS and IP paeonol 50 mg/kg.

### 2.4. Assessment of Histopathological Changes in LPS-Induced ALI Rats

The right lung of the rat was fixed with 10% paraformaldehyde by trachea infusion and embedded with paraffin. Hematoxylin and eosin (H&E) staining was conducted with 4 *μ*m tissue slides. The lung injury was assessed with the modified scoring system described by Kristof et al. [13]. In short, two pulmonologists, who were blind to the treatment, randomly selected 10 fields of lung sections from 3 lobes of right lung tissue per rat and read and scored the damaged levels according to the presence and extent of interstitial cellular infiltration, alveolar protein exudation, and tissue hemorrhage by using microscope at 200x magnification. Normal presentation in each category was scored as 0, and the most severe damaged in each category was scored as 3. If the alveolar space is full with inflammatory cells (such as neutrophils and macrophages), it is denoted as grade 3. Grade 1 reflects just a few inflammatory cell infiltrated. [Less than half of the alveoli are filled denotes as grade 2]. Similarly, the extent of pulmonary infiltration of protein exudation (reddish-stained) and alveolar hemorrhage in the lung is determined by this way. The sum of each category from 10 different microscopic fields is denoted as final damaged score in a rat. The total lung injury score of each rat was determined as the sum of three individual scores of alveolar cellularity, protein exudation, and tissue hemorrhage. If the interpretations of the two physicians are quite different, the slides will be checked by a pathologist.

### 2.5. Measurement of MPO Activity

The level of lung MPO, a marker of neutrophil infiltration [2, 15], was measured. Right lung tissue (1 gm) was homogenized in 1.5*∼*4.0 N-ethylmaleimide (Sigma) for 30 s on ice, and the homogenate was centrifuged at 12,000 g for 30 min at 4°C. The pellet was re-suspended in 4 mL of potassium phosphate buffer (50 mM pH 6.0) with 0.5% hexadecyltrimethylammonium bromide (HTAB). The sample was sonicated for 30*∼*90 s on ice once more. The sample was then incubated at 60°C for 2 h to deactivate tissue MPO inhibitor and then was centrifuged at 12,000 g for 10 min. The supernatant fluids containing MPO were incubated in a 50 mM potassium phosphate buffer (KH_2_PO_4_, pH 6.0) buffer containing the substrate H_2_O_2_ (1.5 M) and o-dianisidine dihydrochloride (167 mg/mL; Sigma-Aldrich, USA) for 30 min. The enzymatic activity was determined spectrophotometrically by measuring the change in absorbance at 460 nm by using a 96-well plate reader. 

### 2.6. Western Blot for the Measurement iNOS

Frozen right lung samples were defrosted and were homogenized with lysis buffer (RIPA with protease inhibitor, with a ratio of lung tissue/lysis buffer equal to 100 mg/400 mL). The homogenate was sonicated and centrifuged at 12,000 ×g for 30 min at 4°C. The supernatant was collected, and its protein concentration was measured by conducting Bradford protein assay (Bio-Rad, Hercules, CA, USA). Aliquots (40 *μ*g) of protein from each lung tissue supernatant were electrophoresed on a sodium dodecyl sulfate-polyacrylamide gel electrophoresis (SDS-PAGE) (7%) for 3 h at 100 V. The protein samples were transferred onto a nitrocellulose membrane (Amersham, USA). The membrane was then probed with polyclonal rabbit anti-mouse iNOS antibody (1 : 500 dilution, GTX213110-01, GeneTex, CA, USA) at 4°C overnight, with appropriate secondary antibodies. After three washes with TPBS, blots enhanced with brown color indicated the expression of iNOS.

### 2.7. Cell Counting and Measurement of Protein, Pro-Inflammatory Cytokines, and Thrombin-Anti-Thrombin Complexes (TATC), Plasminogen Activator Inhibitor (PAI-1) in BALF in LPS-Induced ALI Rats

The right main bronchus was ligated, and then a cannula was inserted into the left lung through the left main bronchus. The BALF was obtained with three aliquots of 8 mL sterile saline instilled up to a total volume of 24 mL, and withdrawn three times each, with final fluid recovery being around 20 mL. The BALF from each animal was recovered and was then passed through a mesh (200 *μ*m) to remove the mucus, followed by centrifugation (1500 ×g, 4°C) for 15 min. The sediment cells were resuspended in 2 mL PBS. Erythrocytes were lysed using cold water and a hypertonic recovery solution (10x HBSS). The erythrocyte-free cell suspension was then washed once by 1x PBS and was used for total cell count. Finally, 2 × 10^5^ BALF-derived cells were evenly distributed onto a slide by cytospin and then stained with Liu's stain for 2 min, before the neutrophils were counted under the microscope.

The resulting supernatants were stored at  −70°C until the analysis stage. Cell counts and protein concentration were measured by Bradford protein assay (Bio-Rad, Hercules, CA, USA).

The proinflammatory cytokines in the BALF, such as TNF-*α* (BMS622MST, BenderMedsystem), IL-1*β* (BMS630, BenderMedsystem), IL-6 (BMS625MST, BenderMedsystem), and IL10 (14-8101-62, eBioscience), were measured using commercially available ELISA and in accordance with the manufacturer's protocol (Assay Designs, Inc. MI, USA). Specifically, this entailed pipetting the samples of BALF supernatant (100 *μ*L) and the standards into the cytokine antibody precoated microplate wells. The plates were tapped gently to mix the contents and incubated at 37°C for 60 min. The contents of the wells were removed, and the wells were washed three times with 400 *μ*L of wash solution. Human cytokine antibodies (100 *μ*L) were added into each well and incubated at 37°C for 60 min. We emptied the contents of the wells and again applied 400 *μ*L of wash solution to every well three times. Horseradish peroxidase (100 *μ*L) was added to conjugate the human cytokine antibody. After incubation at 37°C for 30 min, 100 *μ*L of Substrate Solution TMB (tetramethylbenzidine) was added into each well for 15 min at room temperature.

Color development was stopped by adding Stop Solution (100 *μ*L, 1 N solution of hydrochloric acid in water). Colorimetric determination was read at absorbance of 450 nm (Microplate Reader BIO-RAD Laboratories, CA, USA). Serial dilution of each original recombination human cytokine was performed to draw a standard curve with linear range from 0 to 500 pg/mL. Concentrations of BALF cytokines were measured by comparing the absorbance of the standards, and expressed as picograms per milliliter (pg/mL).

TATC was used as a measured of coagulation through the tissue factor pathway; high TATC level reflects the activation of the coagulation system [42, 43]. TATC was measured using the TATC enzyme-linked immunosorbent assay Micrognost kit, in accordance with the manufacturer's instructions (Assay Max human thrombin-antithrombin TAT complex ELISA kit, ET1020-1 Lot no. 1259916R1). The level of PAI-1 antigen in BALF was measured by Rat PAI-1 total antigen assay ELISA kit (Catalog no. RPAIKT-TOT, Molecular Innovations, MI, USA) according to the manufacturer's instructions.

### 2.8. Measurement of Lung Weight Gain (LWG) in LPS-Induced ALI Rats

The other 24 SD rats were divided into four groups of 6 rats as follows:

1. PBS-DMSO group,
2. PBS-Paeonol group,
3. LPS-DMSO group, and
4. LPS-Paeonol-50 group.

The LPS-induced ALI animal model was the same as described above. At 16 h after inoculation, all rats were sacrificed with CO_2_ asphyxiation. The total lung (including bilateral trachea) was separated from the chest cavity of the rat, and the body fluid coating the removed lung was wiped gently. The bilateral main bronchus was cut from the hilum area. The residual whole lung was placed on silver paper on an electronic scale, and the weight was recorded as whole lung weight (WLW). The whole lung on the silver paper was placed into an oven at 60°C for 48 h. Then the sample was taken out of the oven and weighed again on an electronic scale, with the weight being recorded as dry lung weight (DLW). The WLW minus the DLW indicated the net lung weight gain (LWG). LWG reflected the net lung edema status after manipulation [44].

### 2.9. Statistical Analysis

All data were expressed as mean ± SEM, using the data from at 6 rats except iNOS (*n* = 4). Statistical analysis of the data was conducted using Prism 3.02 software (GraphicPad Software Inc. CA, USA), via one-way ANOVA for multiple comparisons (post hoc Tukey test). Results with *P* < 0.05 were considered statistically significant.

## 3. Results

### 3.1. Effects of Paeonol on Histopathological Changes of Lung in LPS-Induced ALI Rats

**Figure 1:**
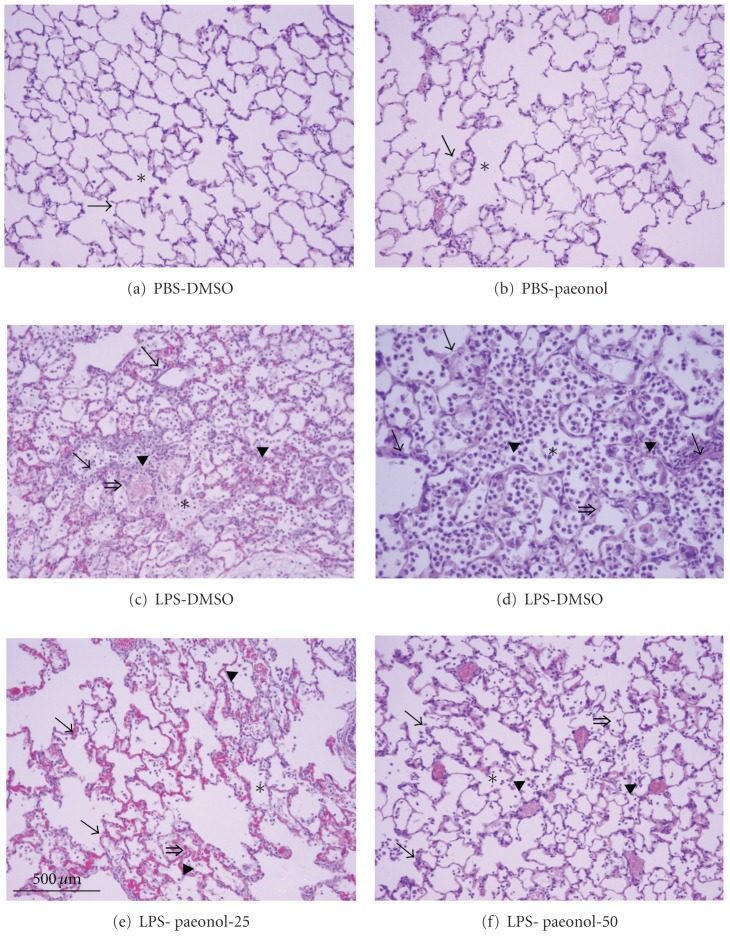
Effects of paeonol on LPS-induced histopathological damage in rats. Histopathological damage developed 16 h after intratracheal administration of LPS (16 mg/kg). The alveolar spaces (∗) are filled with a mixed mononuclear/neutrophilic infiltrate (▸), red blood cells (*⇒*), and protein exudation. The alveolar walls (→) are thickened and edematous. Note the presence of cellular debris and proteinaceous material in the air spaces in the lung tissue of the LPS-DMSO group (c) with 200x; (d) with 400x. No prominent neutrophil infiltration, red blood cells or protein exudates were seen in the PBS-DMSO group (a) and PBS-paeonol group (b). The infiltration of neutrophil, red blood cells and protein exudation was reduced in the LPS-paeonol-25 group (e) and LPS-paeonol-50 group (f).

In the PBS-DMSO and PBS-paeonol groups, the alveolar space was not prominent fluid, protein accumulation, and the infiltration of inflammatory cells and red blood cells (Figures 1(a) and 1(b)). In contrast, the alveolar spaced presented with fluid and protein accumulation, large amounts of inflammatory cells, and red blood cell infiltration at 16 h after LPS IT instillation (16 mg/kg) (Figures 1(c) and 1(d)). Paeonol treatment with 25 mg/kg or 50 mg/kg, administered after the LPS challenge, markedly attenuated inflammatory cell infiltration and alveolar wall thickening and diminished alveolar hemorrhage and edema (Figures 1(e) and 1(f)). A semiquantitative analysis of the histopathological scores of the rats' lungs is presented in Table 1.

### 3.2. Effects of Paeonol on RT in LPS-Induced ALI Rats

**Figure 2:**
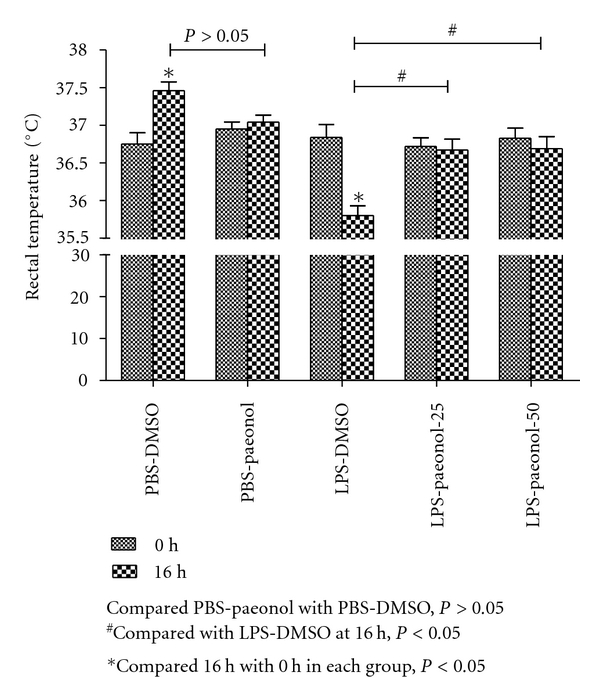
Effect of paeonol on rectal temperature (RT) changes after LPS-induced acute lung injury in rats. In the PBS-DMSO group, RT was greater at 16 h than at 0 h; in the LPS-DMSO group, RT was lower at 16 h than at 0 h. *P < 0.05 compared with 0 h. ^#^ *P* < 0.05 compared with 16 h.

**Figure 3:**
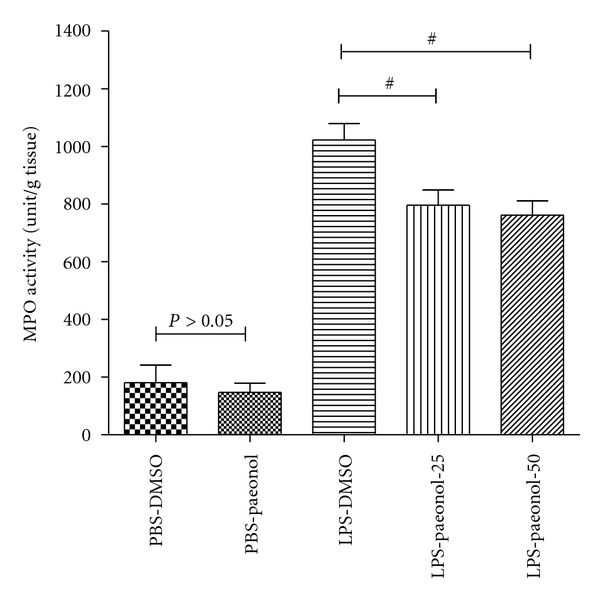
Effect of paeonol on myeloperoxidase (MPO) activity in LPS-induced acute lung injury in rats. The MPO activity was similar between PBS-DMSO and PBS-paeonol groups (*P* > 0.05), whereas the MPO activity was lower in the LPS-paeonol-25 and in the LPS-paeonol-50 groups than LPS-DMSO group. ^#^ *P* < 0.05 compared with LPS-DMSO.

The RT in the LPS-DMSO group at 16 h after IT administration was lower than at baseline (0 h), with the difference being statistically significant (*P* < 0.05) (Figure 2). In contrast, for the PBS-paeonol and LPS-paeonol-50 groups, the RT at 16 h was similar to the baseline temperature (both *P* > 0.05) (Figure 2). The RT in the PBS-DMSO group at 16 h after IT administration was higher than at baseline (*P* < 0.05) (Figure 3).

### 3.3. Effect of Paeonol on MPO Activity and iNOS Expression

The MPO activity of lung tissue was greater in the LPS-DMSO, LPS-paeonol-25, and LPS-Paeonol-50 groups relative to the PBS-DMSO and PBS-paeonol groups, at 16 h after IT administration (all *P* < 0.05) (Figure 3). The MPO activity of lung tissue was lower in the LPS-paeonol*-*25 and LPS-paeonol*-*50 groups relative to the LPS-DMSO group, at 16 h after IT administration (both *P* < 0.05) (Figure 3).

**Figure 4:**
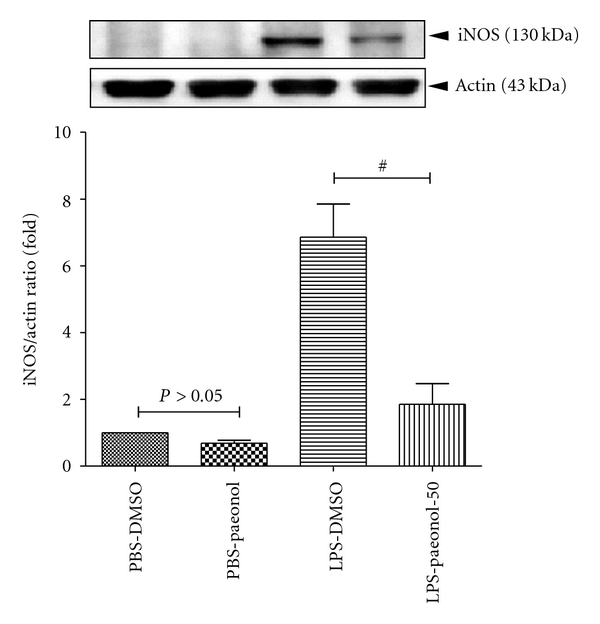
Effect of paeonol on inducible nitric oxide synthase (iNOS) activity in LPS-induced acute lung injury rats. The iNOS expression was similar between PBS-DMSO and PBS-paeonol groups (*P* > 0.05), whereas the iNOS expression was lower in the LPS-paeonol-50 groups than LPS-DMSO group. ^#^ *P* < 0.05 compared with LPS-DMSO.

The iNOS expression of lung tissue was greater in the LPS-DMSO group relative to the PBS-DMSO and PBS-paeonol groups, at 16 h (both *P* < 0.05) (Figure 4). The iNOS expression of lung tissue was lower in the LPS-paeonol-50 group than in the LPS-DMSO group at 16 h after IT administration (*P* < 0.05) (Figure 4).

### 3.4. Effects of Paeonol on Leukocyte Accumulation and Protein Exudation

**Figure 5:**
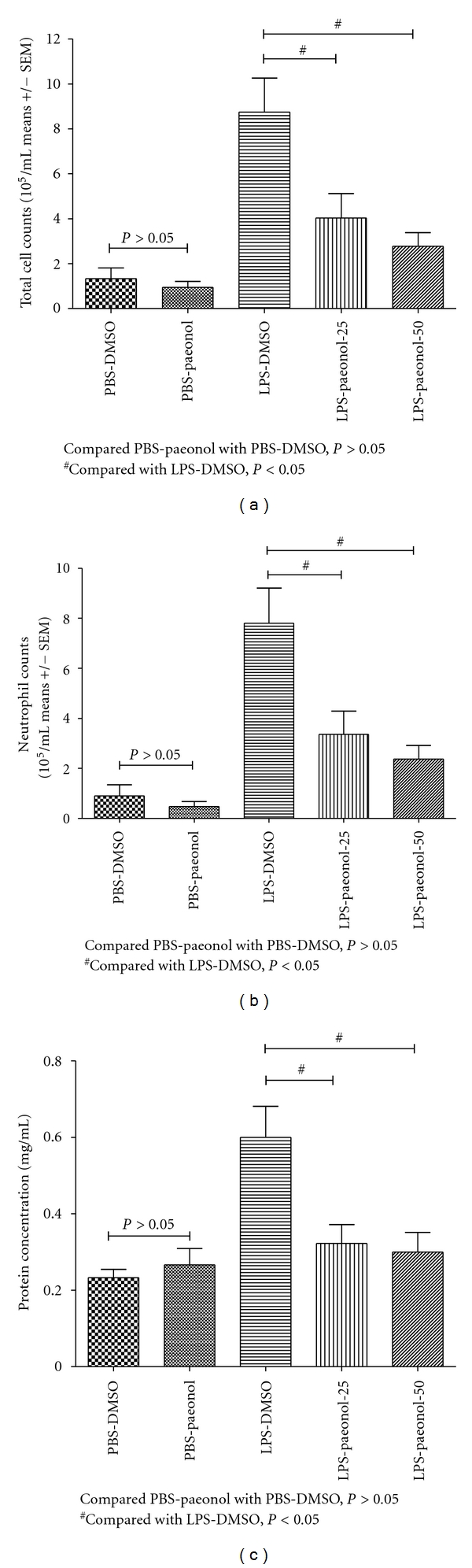
Effect of paeonol on leucocyte cell count and protein exudation of BALF in LPS-induced acute lung injury in rats. The total cell count (a), neutrophil count (b), and protein exudation (c) of BALF was similar between PBS-DMSO and PBS-paeonol groups (all *P* > 0.05), whereas the total cell count, neutrophil count, and protein exudation of BALF was lower in the LPS-paeonol-25 and in the LPS-paeonol-50 groups than LPS-DMSO group. ^#^ *P* < 0.05 compared with LPS-DMSO.

The total leukocyte counts of BALF were greater in the LPS-DMSO group relative to the PBS-DMSO and PBS-paeonol groups, at 16 h after IT administration (both *P* < 0.05) (Figure 5(a)). The total leukocyte counts of BALF were lower in the PBS-paeonol-25 and PBS-paeonol-50 groups than in the LPS-DMSO group at 16 h (both *P* < 0.05) (Figure 5(a)).

The total PMN counts of BALF were greater in the LPS-DMSO group than in the PBS-DMSO and PBS-paeonol groups, at 16 h after IT administration (both *P* < 0.05) (Figure 5(b)). The total PMN counts of BALF were lower in the LPS-paeonol-25 and LPS-paeonol 50 groups than in the LPS-DMSO group at 16 h (both *P* < 0.05) (Figure 5(b)).

The protein concentration of BALF was greater in the LPS-DMSO group than in the PBS-DMSO and PBS-paeonol groups at 16 h after IT administration (both *P* < 0.05; Figure 5(c)). The protein concentration of BALF was lower in the LPS-paeonol-25 and in the LPS-paeonol-50 groups than these in the LPS-DMSO group at 16 h (both *P* < 0.05; Figure 5(c)).

### 3.5. Effect of Paeonol on the Expression of Pro-Inflammatory Cytokines of BALF

**Figure 6:**
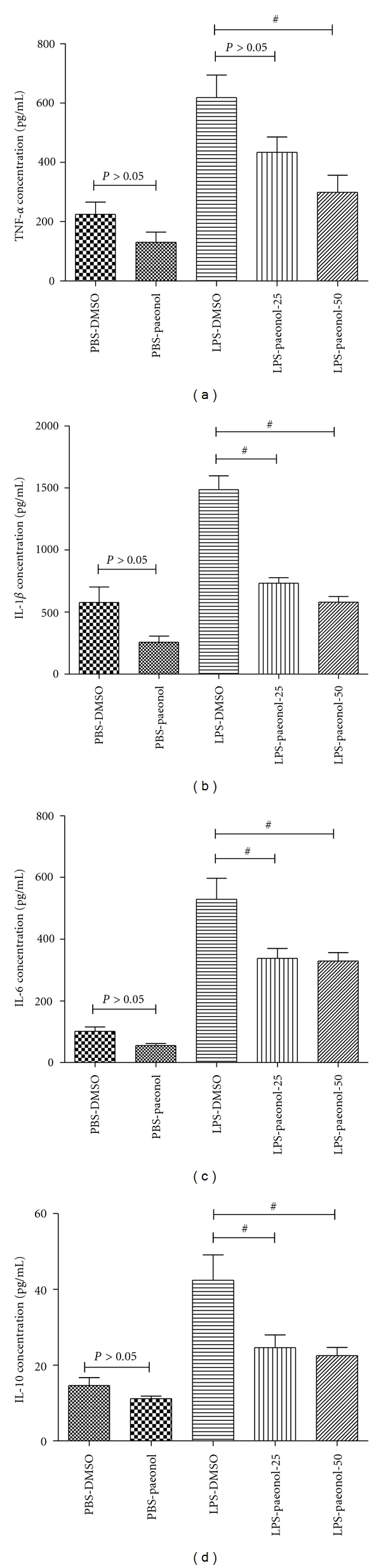
Effect of paeonol on tumor necrosis factor-*α* (TNF-*α*), interleukin-1*β* (IL-1*β*), IL-6, and IL-10 concentrations of BALF in LPS-induced acute lung injury in rats. The concentrations of TNF-*α* (a), IL-1*β* (b), IL-6 (c), and IL-10 of BALF were similar between PBS-DMSO and PBS-paeonol group (all *P* > 0.05), whereas the concentrations of TNF-*α*, IL-1*β*, IL-6, and IL-10 of BALF were lower in the LPS-paeonol-25 and in the LPS-paeonol-50 groups than LPS-DMSO group. ^#^ *P* < 0.05 compared with LPS-DMSO.

The TNF-*α*  expression of BALF was greater in the LPS-DMSO group than in the PBS-DMSO and PBS-paeonol groups at 16 h after IT administration (both *P* < 0.05) (Figure 6(a)). The TNF-*α*  expression of BALF was lower in the LPS-paeonol-50 group than in the LPS-DMSO group at 16 h (*P* < 0.05) (Figure 6(a)). However, TNF-*α* expression in the LPS-DMSO group was similar to that of the LPS-paeonol-25 group (*P* > 0.05) (Figure 6(a)).

The IL-1*β*  expression of BALF was greater in the LPS-DMSO group than in the PBS DMSO and PBS-paeonol groups at 16 h (both *P* < 0.05) (Figure 6(b)). The IL-1*β*  expression of BALF was lower in the LPS-paeonol-25 and the LPS-paeonol-50 groups than in the LPS-DMSO group at 16 h (both *P* < 0.05) (Figure 6(b)).

The IL-6 expression of BALF was greater in the LPS-DMSO group than in the PBS DMSO and PBS-paeonol groups at 16 h (both *P* < 0.05) (Figure 6(c)). The IL-6 expression of BALF was lower in the LPS-paeonol-25 and LPS-paeonol-50 groups than in the LPS-DMSO group at 16 h (both *P* < 0.05; Figure 6(c)).

The IL-10 expression of BALF was greater in the LPS-DMSO group than in the PBS DMSO and PBS-paeonol groups at 16 h (both *P* < 0.05) (Figure 6(c)). The IL-10 expression of BALF was lower in the LPS-paeonol-25 and LPS-paeonol-50 groups than in the LPS-DMSO group at 16 h (both *P* < 0.05; Figure 6(d)).

### 3.6. Effect of Paeonol on the TATC and PAI-1 Concentration of BALF in LPS-Induced ALI Rats

**Figure 7:**
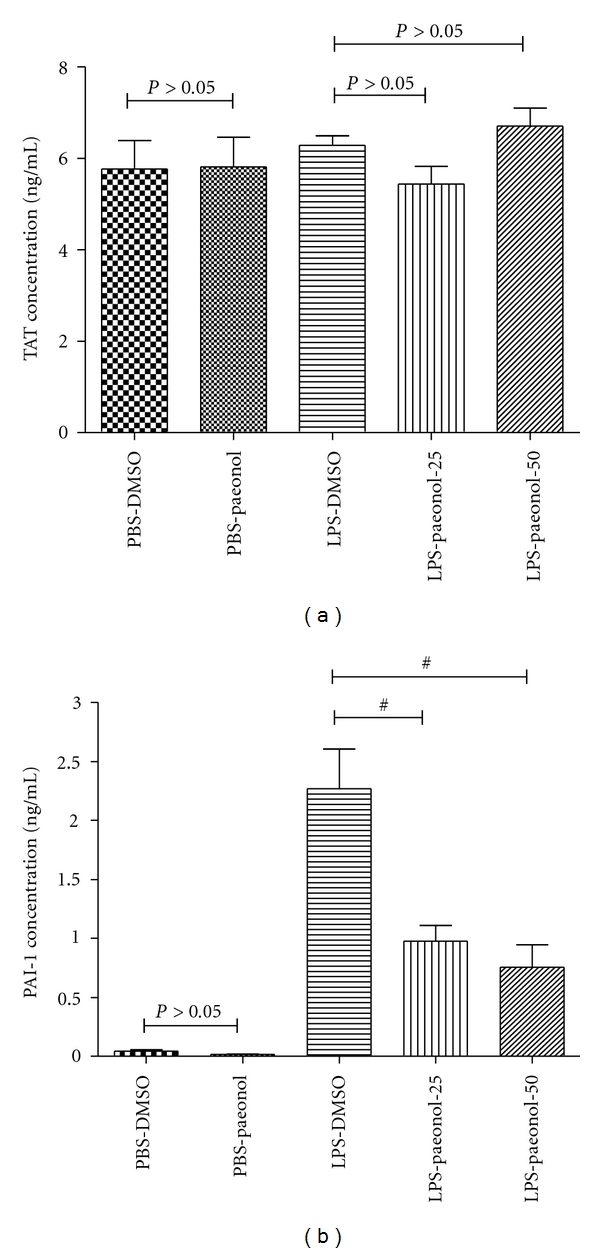
Effect of paeonol on thrombin-anti-thrombin complexes (TATC) and plasminogen activator inhibitor (PAI-1) of BALF in LPS-induced acute lung injury in rats. The concentrations of TATC in BALF were similar between PBS-DMSO and PBS-paeonol groups (*P* > 0.05), between LPS-DMSO and LPS-paeonol-25 groups (*P* > 0.05), and between LPS-DMSO and LPS-paeonol-50 groups (*P* > 0.05) (a); The PAI-1 was similar between PBS-DMSO and PBS-paeonol groups (*P* > 0.05), whereas the PAI-1 was lower in the LPS-paeonol-25 and in the LPS-paeonol-50 groups than LPS-DMSO group. ^#^ *P* < 0.05 compared with LPS-DMSO.

The TATC concentration of BALF in the LPS-DMSO group was similar to that of the PBS-DMSO and PBS-paeonol groups at 16 h after IT administration (both *P* > 0.05) (Figure 7(a)).

The TATC concentration of BALF in the LPS-DMSO group was also similar to that of the LPS-paeonol-25 and LPS-paeonol-50 groups at 16 h (both *P* > 0.05) (Figure 7(a)).

The PAI-1 concentration of BALF was greater in the LPS-DMSO group than in the PBS-DMSO and PBS-paeonol groups at 16 h (both *P* < 0.05) (Figure 7(b)). The PAI-1 concentration of BALF was lower in the LPS-paeonol-25 and LPS-paeonol*-*50 groups than in the LPS-DMSO groups at 16 h (both *P* < 0.05) (Figure 7(b)).

### 3.7. Effects of Paeonol on LWG in LPS-Induced ALI Rats

**Figure 8:**
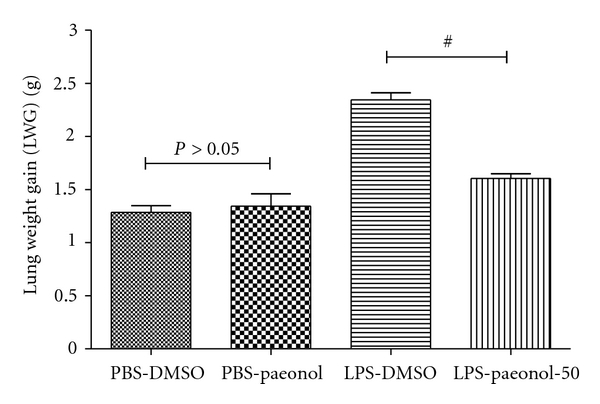
Effect of paeonol on lung weight gain (LWG) in LPS-induced acute lung injury in rats. The LWG was similar between PBS-DMSO and PBS-paeonol groups (*P* > 0.05), whereas the LWG was lower in the LPS-paeonol-50 groups than LPS-DMSO group. ^#^ *P* < 0.05 compared with LPS-DMSO.

Lung weight gain was greater in the LPS-DMSO and LPS-paeonol-50 groups compared to the PBS-DMSO group (both *P* < 0.05) at 16 h after LPS IT administration (Figure 8). The LWG in the PBS-paeonol and LPS-paeonol-50 groups was lower than that of the LPS-DMSO group (both *P* < 0.05) (Figure 8).

## 4. Discussion

Our results indicated that paeonol attenuated histopathological damage scores in the LPS-induced ALI rat model. Paeonol reduced the activity of MPO and the expression of iNOS, and the concentration of PAI-1 in lung tissue, and also reduced LWG. In addition, *Paeonol* reduced TNF-*α*, IL-1*β*, IL-6, and PAI-1 concentrations in the BALF. Therefore, we suggest that paeonol protects the lung in LPS-induced ALI rats, and that the effect occurs through the anti-inflammation and anti-coagulation properties of paeonol. MPO activity is a marker of PMN neutrophil [2, 15], whereas iNOS increases NO production and mediates lung inflammation [11–14]. The proinflammatory cytokines TNF-*α*, IL-1*β*, and IL-6 mediate the inflammatory cascade [8, 9, 12, 45]. PAI-1 may represent an activation of fibrinolysis [18, 21, 46]. In addition, we used paeonol 25 mg/kg and 50 mg/kg in the present study, if paeonol 100 mg/kg such as Du et al. (2010) [47] whether or not produce greater anti-inflammatory and anti-coagulative effects need further study. 

Our results remain one question unanswered that is the levels of IL-10, is an anti-inflammatory cytokine, were lower in the LPS-paeonol-25 and LPS-paeonol-50 groups than these in the LPS-DMSO group were seemly contrast to the results of Chou (2003) [36]. One possible explanation was that the levels of IL-10 reached a peak at 16–24 h [48], whereas the IL-10 was measured at 16 h after LPS administration, therefore, the maximal levels could not obtain in the present study. This explanation needs further study.

Several animal models have been developed to mimic the pathophysiology of ALI-following LPS exposure [5–7]. In the present study, we have produced a constant and reproducible rat model to investigate the treatment effects of paeonol in ALI by delivering LPS directly into airway in our laboratory [37, 38]. The pulmonary histopathological changes of LPS-induced ALI are characterized by PMN accumulation, disruption of epithelial integrity, interstitial edema, and leakage of a large amount of protein into the alveolar spaces [1–4]. The results of the present study indicated that paeonol treatment may reduce the drop in RT, which typically follows LPS challenge. Hypothermia may be an early phenomenon of sepsis; thus, paeonol might play a critical role in preventing sepsis. In contrast, LWG may indicate the degree of lung edema, which Paeonol may also help reduce in the LPS-induced ALI model.

It has been well documented in both animal models and humans that LPS stimulates iNOS expression and NO over-production, which damages lung tissue via peroxynitrite formation [12, 14, 46, 49, 50]. One recent report showed that paeonol inhibits LPS-induced iNOS expression through deactivating the mitogen-activated protein kinase (MAPK) in RAW 264.7 macrophage-like cells [35]. However, the effect of paeonol in the ALI rat model remains unclear. In this study, we demonstrated that paeonol significantly attenuates iNOS expression after a rat has been exposed to LPS challenge, suggesting that one anti-inflammatory mechanism of paeonol protects the lung from damage.

High levels of proinflammatory cytokines such as TNF-*α*, IL-1*β*, and IL-6 play a central role in the initiation and propagation of the inflammatory cascade [8, 9, 12, 45]. A large amount of PMN is recruited from peripheral blood into the lung, producing large amounts of MPO and reactive oxygen derivatives. The result is a cascade-like response, and tissue damage [2, 15, 49, 51]. In line with these theories, we found that after instilling LPS by IT, large amounts of proinflammatory cytokines were expressed in the BALF in rat lung parenchyma, with enhanced activity of MPO. Paeonol IP injections significantly suppressed the expression of TNF-*α*, IL-1*β*, and IL-6 in rat BALF; this also reduced LPS-induced pulmonary parenchymal MPO activity. As expected the number of PMNs in BALF had decreased 16 h after treatment, suggesting a mechanism by which paeonol attenuates the LPS-induced ALI model.

The hallmark of ALI is the deposition of fibrin and platelet plugs, which induce the occlusion of microvasculature of the alveolar space [3, 52]. As excessive fibrin is deposited within the airways, neutrophils and fibroblasts may further be activated. This situation compromises gas exchange and pulmonary endothelial integrity, decreases alveolar fluid clearance, and finally leads to pulmonary microcirculation damage and death [16, 52, 53].

Pulmonary coagulopathy is now accepted as a target in therapeutic studies of acute lung injury or pneumonia [16–18, 21, 27]. The available data suggest that high levels of proinflammatory cytokines (such as TNF, IL-1, and IL-6) may activate the coagulation cascade by stimulating TF expression. High levels of these cytokines may also attenuate fibrinolysis by stimulating the release of PAI [16, 18–21]. Our data showed no significant difference in TATC level of BALF after paeonol treatment. However, the PAI-1 levels in BALF were significantly decreased in the paeonol treatment group, suggesting a strong anti-fibrinolytic effect of paeonol in ALI-induced coagulopathy. Since proinflammatory cytokines serve as potent regulators of macrophage PAI-1 production in ALI, it remains unclear whether the anti-fibrinolysis effect of paeonol occur via inhibiting PAI-1 expression directly, or is perhaps the consequence of anti-proinflammatory cytokines. Further experiments are needed to clarify the target effects on cells, and the causal relationship between anti-inflammation and anti-coagulation effects of paeonol.

In conclusion, the results of the current study demonstrated that paeonol protects against lung tissue damage in the LPS-induced ALI model. This finding suggests that these effects are because of the anti-inflammatory and anti-coagulant properties of paeonol. Thus paeonol may be a potential therapeutic reagent for treating ALI in the future.

## Figures and Tables

**Table 1:** Effect of paeonol on the histopathological scores in lipopolysaccharide-induced acute lung injury.

	Cellularity	Protein exudation	Hemorrhage	Scores
PBS-DMSO	7.2 ± 1.0	3.8 ± 0.6	8.1 ± 1.8	19.0 ± 2.5
PBS-paeonol	4.5 ± 0.2	2.7 ± 0.6	7.8 ± 0.7	14.9 ± 1.2
LPS-DMSO	18.0 ± 0.7	10.9 ± 0.8	16.4 ± 1.0	45.4 ± 1.0
LPS-paeonol-25	10.8 ± 0.9^#^	4.5 ± 0.8^#^	11.5 ± 1.5	26.8 ± 2.4^#^
LPS-paeonol-50	9.1 ± 1.2^#^	4.1 ± 0.4^#^	10.5 ± 1.5^#^	23.7 ± 2.9^#^

Data are presented as mean ± SEM; PBS-DMSO: PBS-DMSO group; PBS-paeonol: PBS-paeonol group; LPS-DMSO: LPS-DMSO group; LPS-paeonol-25: LPS-paeonol-25 mg/kg group; LPS-paeonol-50: LPS-paeonol-50 mg/kg group; ^#^ *P* < 0.05 compared with LPS-DMSO; *n* = 6.
